# Supplementary material for: Efficient Reuse of Natural Language Processing Models for Phenotype-Mention Identification in Free-text Electronic Medical Records: A Phenotype Embedding Approach
Source: JMIR Med Inform. 2019 Dec 17;7(4):e14782. doi: 10.2196/14782 (PMC6938594; doi:10.2196/14782)

## **Multimedia Appendix 1: User interface and model performances of phenotype NLP models**

### **MA****1. The user interface for interactively adapting a generic NLP model for a research study**

Figure MA1(a) SemEHR provides a semantic search interface to access generic/baseline natural language processing results.


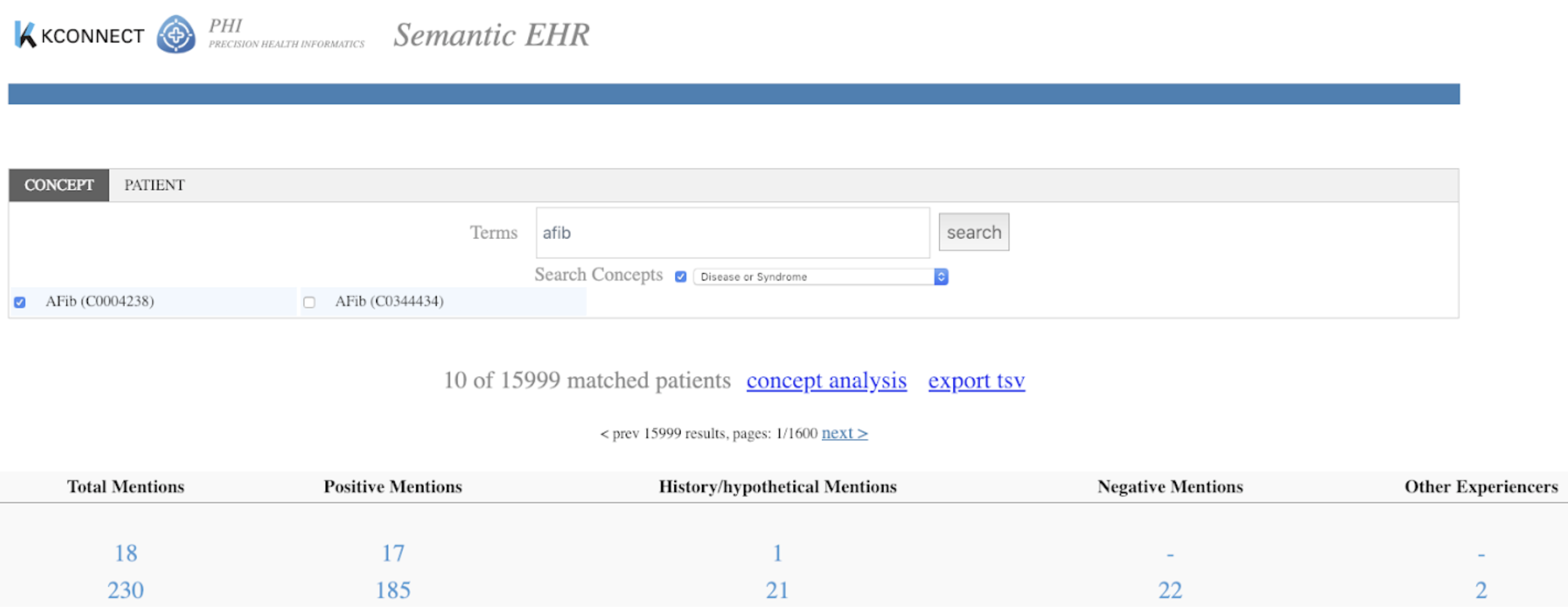


Figure MA1(b) Feedback buttons are provided along with NLP annotations in the search results. Feedbacks provided by a researcher will be used to populate a dedicated and better model for her research study. Meanings of the buttons: posM - positive mention; hisM - history mention; hypoM - hypothetical mentions; negM - negated mentions; otherM - other experiencer mention; wrongM - not a phenotype mention.


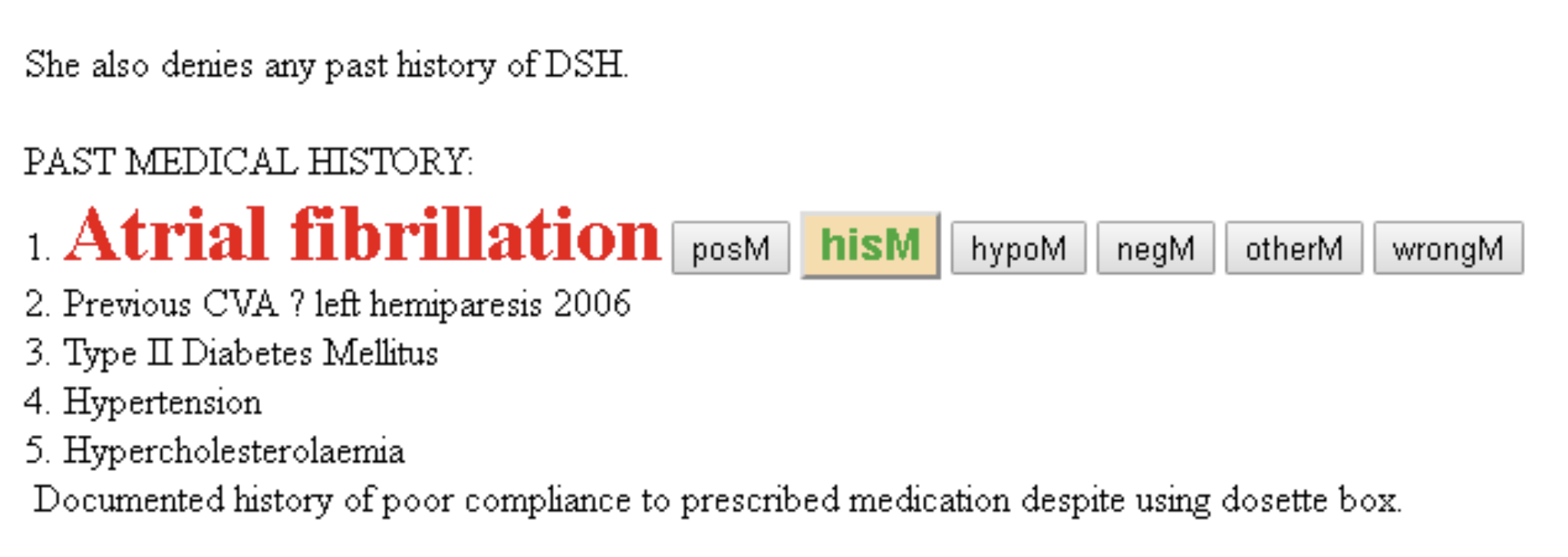


### **MA****2. Performances and feedbacks of 23 phenotype identification models on SLaM EHR data**

Figure MA2 (a) Statistics of identified physical conditions: condition mention - mentions that are related to the condition; positive mention - mention of a condition that the patient suffered from; num concepts - number of sub-concepts (e.g. TIA, Brain haemorrhage) that constitute a physical condition (e.g. stroke).


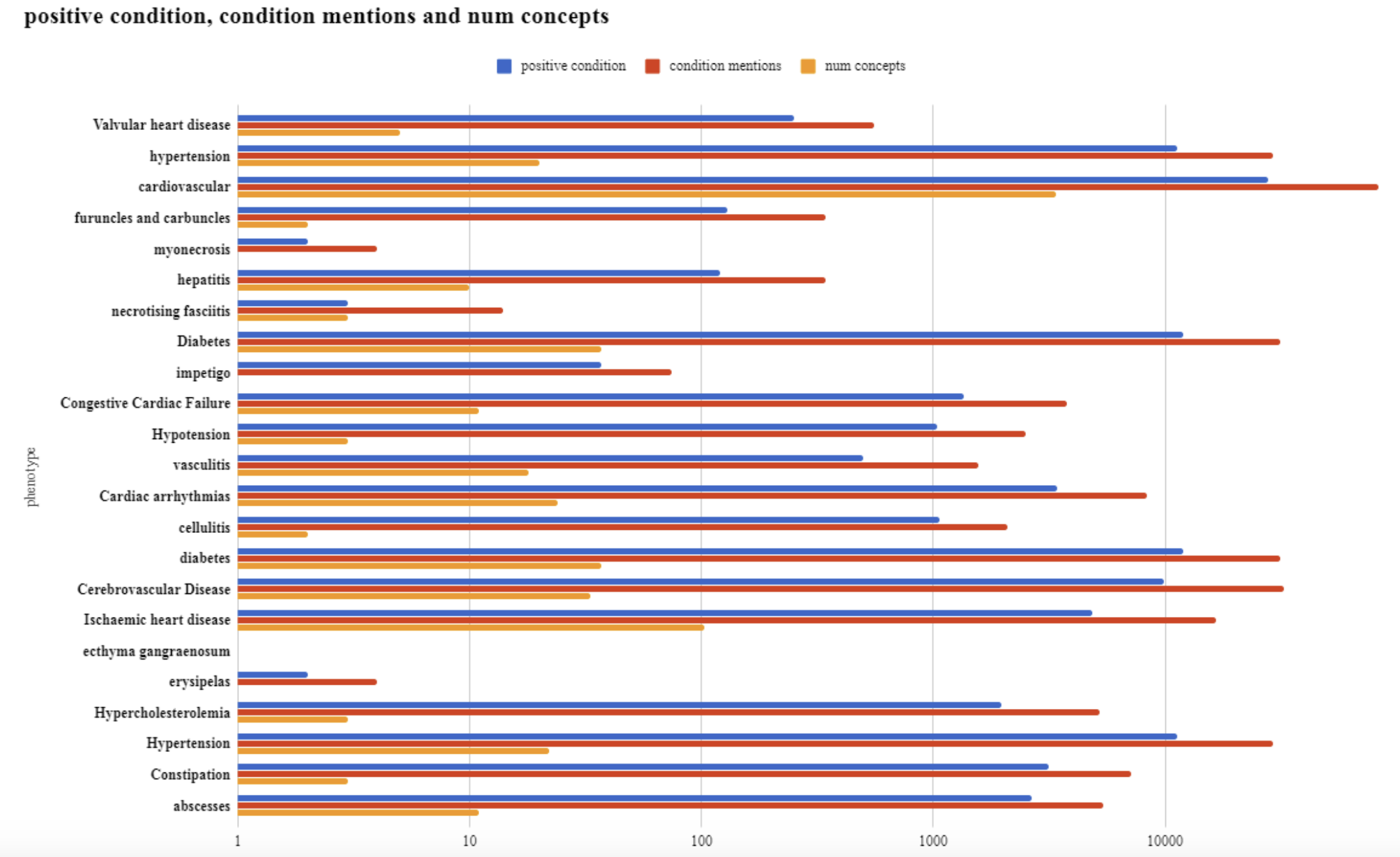


Figure MA2 (b) Mention accuracy (the accuracy of NLP tool identified condition mentions) of 23 physical conditions.


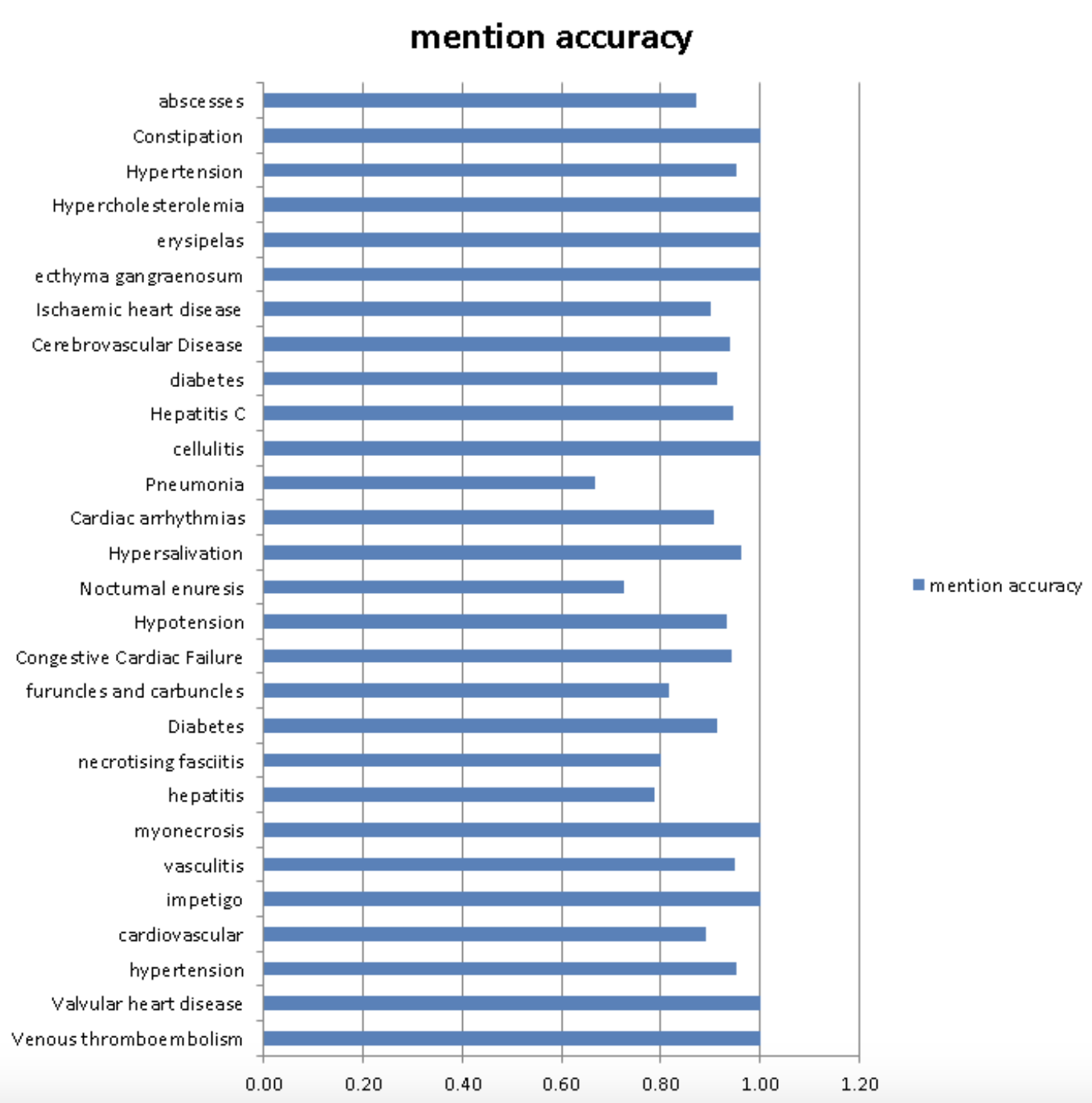


Figure MA2 (c) The numbers of feedbacks needed to iteratively train a good model for a physical condition (shows the top 10 most validated conditions).


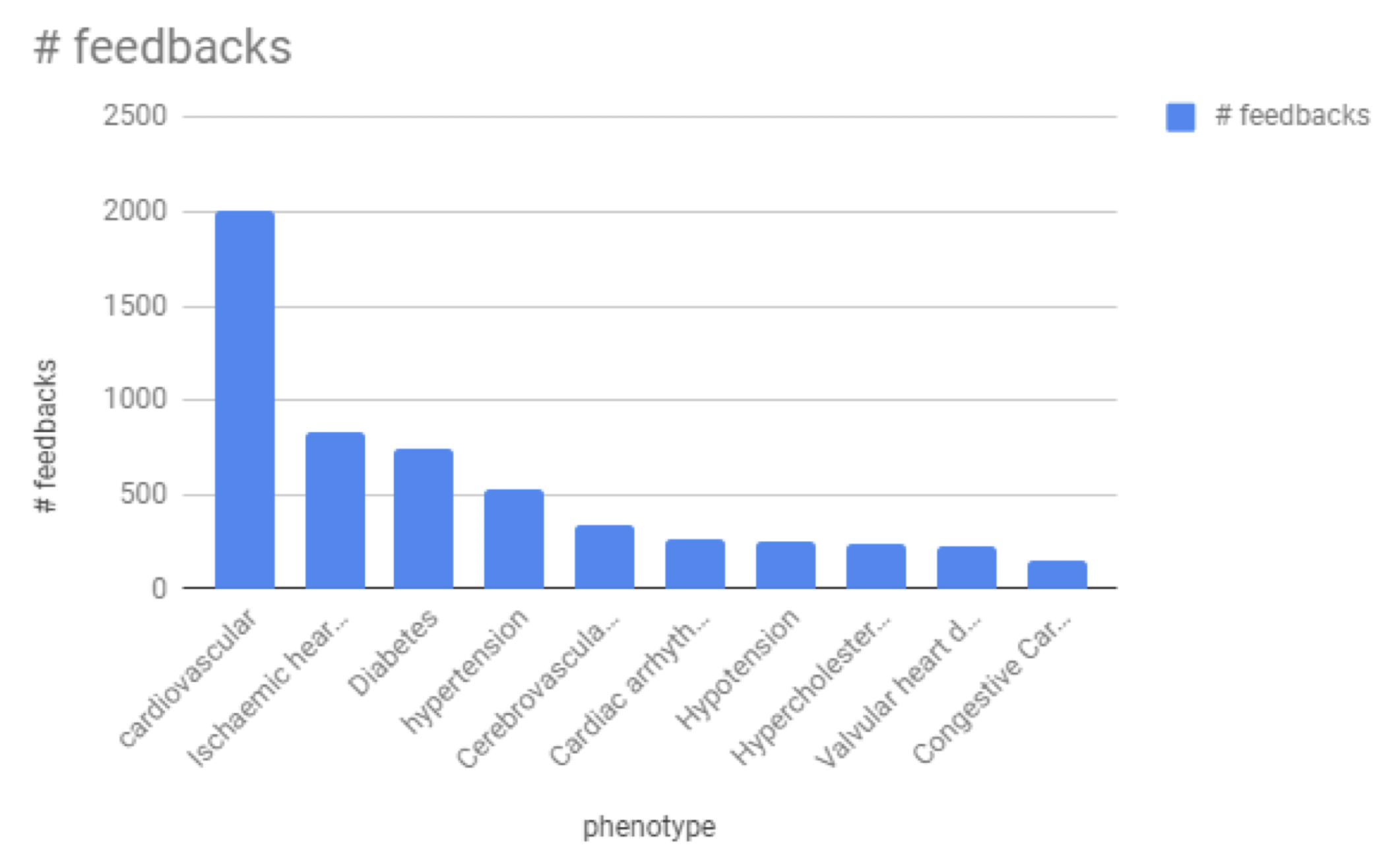

Supplement: Multimedia Appendix 1 [file medinform_v7i4e14782_app1.docx]
