# Supplementary material for: Efficient Reuse of Natural Language Processing Models for Phenotype-Mention Identification in Free-text Electronic Medical Records: A Phenotype Embedding Approach
Source: JMIR Med Inform. 2019 Dec 17;7(4):e14782. doi: 10.2196/14782 (PMC6938594; doi:10.2196/14782)
Supplement: Multimedia Appendix 2 [file medinform_v7i4e14782_app2.docx]

## **Multimedia Appendix 2: Proof of Theorem 1**

| *Proof.* Theorem 1 can be proved as follows.   1. Instances of $P_{m}\cap P_{T}$ in $S$ are those mentions whose patterns which are same as m has seen previously. Given the deterministic associations between patterns and m’s performances (item 3 in Assumption 1), their performances can be predicted with high confidence using prior knowledge of m’s performances on these patterns. Therefore, $P_{m}\cap P_{T}$ is a $S_{known}$. 2. $P_{new}=P_{T}-(P_{m}\cap P_{T})$ meets the definition of p-unknown subsets as proved by the following.    1. Apparently, $P_{new}$ meets the criterion 2.a in Definition 1 because all mentions with new patterns are represented by it;    2. Based on item 1 in Assumption 1, each $p\in P_{new}$ is disjoint with others because a mention can only be assigned to one pattern. Therefore, criterion 2.b is met.    3. Item 3 in Assumption 1 embraces criterion 2.c in that it induces performances of $m$ on all mentions of a pattern $p$ can be deduced by observing how it performs on sampled mentions of $p$.    4. Item 2 Assumption 1 assures the fulfilment of criterion 2.d - a small number of patterns.   ☐ |
| --- |
